# Reduced thrombospondin-1 is associated with altered collagen-rich matrix organization in discoid lateral meniscus

**DOI:** 10.3389/fcell.2026.1958871

**Published:** 2026-09-30

**Authors:** Shunjie Yang, Chen Hou, Hui Wang, Li Liu, Jian Li, Naihong Yan, Xiaohe Tian, Gang Chen

**Affiliations:** 1 Department of Radiology, Huaxi MR Research Center, Institute of Radiology and Medical Imaging, West China Hospital, Sichuan University, Chengdu, China; 2 Sports Medicine Center, West China Hospital, Sichuan University, Chengdu, China; 3 Department of Orthopedics and Orthopedic Research Institute, West China Hospital, Sichuan University, Chengdu, China; 4 Department of Ophthalmology and Laboratory of Macular Disease, West China Hospital, Sichuan University, Chengdu, China; 5 The Academy of Chinese Health Risks of West China Hospital, Sichuan University, Chengdu, China; 6 Department of Orthopedic Surgery, West China Hospital, Sichuan University/West China School of Nursing, Chengdu, China; 7 Research Laboratory of Ophthalmology, West China Hospital, Sichuan University, Chengdu, China; 8 The Xiamen Key Laboratory of Psychoradiology and Neuromodulation, West China Xiamen Hospital of Sichuan University, Xiamen, China

**Keywords:** collagen-rich matrix, discoid lateral meniscus, extracellular matrix, meniscal cells, proteomics, spheroids, thrombospondin-1, transcriptomics

## Abstract

Discoid lateral meniscus (DLM) is susceptible to tearing and degeneration, but the molecular basis of its altered extracellular matrix (ECM) remains unclear. We integrated histology, electron microscopy, transcriptomics, quantitative proteomics, and donor-derived cell and spheroid models to investigate the association between thrombospondin-1 (THBS1) and matrix organization in symptomatic DLM. Compared with traumatic morphologically normal lateral meniscus (NLM), DLM tissue contained fewer cells per analyzed image (mean difference, −16.10; 95% confidence interval (CI), −23.49 to −8.71; P < 0.0001); lower type-I and II collagen signals; and thinner, less dense, and less organized collagen fibrils. Cross-omics analysis nominated THBS1 and ECM-receptor interaction, and independent assays confirmed a 6.67-fold lower THBS1 protein abundance in DLM (95% CI, 5.04 to 8.27; P < 0.0001). In donor-derived models, THBS1 knockdown in NLM cells reduced area-confluence trajectories, increased migration, and decreased collagen-rich matrix in 4-week spheroids, whereas THBS1 overexpression in DLM cells produced reciprocal changes; aggrecan was not consistently altered. These data associate reduced THBS1 with altered collagen-rich matrix organization and support a functional contribution *in vitro*. The cross-sectional clinical comparison does not establish chronology, and the spheroid experiments do not demonstrate a fibrillogenesis-specific mechanism.

## 1 Introduction

Discoid lateral meniscus (DLM) is a congenital anomaly of the knee meniscus characterized by an abnormally disc-shaped lateral meniscus that covers an enlarged area of the tibial plateau (Yang et al., 2023). DLM is frequently reported in Asian populations, can be bilateral, and commonly becomes symptomatic after tearing or degeneration (Chen et al., 2016; Bae et al., 2012; Sheasley et al., 2024; Dzidzishvili et al., 2025). Symptomatic DLM causes pain, mechanical symptoms, and early compartmental degeneration, including in children and young adults (Sheasley et al., 2024; Dzidzishvili et al., 2025). Altered shape and instability create abnormal joint stresses (Kinoshita et al., 2022; Yokoe et al., 2023). Histological and ultrastructural studies have also described disorganized collagen architecture, reduced collagen content, and mucoid degeneration (Cui and Min, 2007; Atay et al., 2007; Papadopoulos et al., 2009; Choi et al., 2017). Saucerization or partial meniscectomy, with repair or stabilization when indicated, remains the standard treatment; however, recurrent tears and lateral-compartment osteoarthritis remain concerns (Sabbag et al., 2019; Lee et al., 2018; Logan et al., 2021; Vivekanantha et al., 2025). Whether the matrix phenotype reflects the developmental biology, chronic abnormal loading, degeneration, or a combination of these processes remains unresolved.

The meniscal extracellular matrix (ECM), composed principally of water, type-I and II collagen, proteoglycans, and matricellular glycoproteins, provides tensile strength, compressive resistance, and load distribution (Kuczyński et al., 2025; Pasiński et al., 2023). ECM synthesis, fibril assembly, cross-linking, and turnover must remain balanced to preserve tissue architecture and mechanics (Bonnans et al., 2014; Naba, 2024). Fetal and clinical studies support a developmental component in DLM (Turati et al., 2021; Campbell et al., 2023), and single-cell RNA sequencing has identified DLM cell populations with altered matrix-regulatory programs (Sun et al., 2022). The molecular processes connecting these cellular abnormalities to the weak discoid matrix remain incompletely defined.

We, therefore, integrated tissue histology, electron microscopy, transcriptomics, quantitative proteomics, and functional modeling in primary meniscal cells and three-dimensional spheroids. We focused on thrombospondin-1 (THBS1), a matricellular glycoprotein involved in cartilage biology, ECM organization, and cell–matrix communication (Li et al., 2024; Murphy-Ullrich, 2019; Tan and Lawler, 2009; Adams, 2024). Our primary objective was to determine whether THBS1 abundance is associated with the DLM matrix phenotype and whether THBS1 perturbation alters meniscal-cell behavior and collagen-rich matrix organization. The cross-sectional clinical comparison was used to identify tissue associations, and donor-derived perturbation models were used to assess functional contribution *in vitro*.

## 2 Materials and methods

### 2.1 Patient samples and study design

Meniscal specimens were collected during arthroscopy from 122 patients with symptomatic DLM and 65 patients with isolated traumatic morphologically normal lateral meniscus (NLM) tears at West China Hospital, Sichuan University. Both groups, therefore, comprised symptomatic operative tissues. Patients with Kellgren–Lawrence grade-II or higher osteoarthritis or Outerbridge grade-II or higher cartilage damage were excluded (Kohn et al., 2016; Noyes and Stabler, 1989). Forty specimens (20 per group) were used for histology and microscopy, 30 (15 per group) for RNA sequencing, 30 for proteomics before quality control, 12 (six per group) for molecular validation, and six (three per group) for cell experiments. One DLM proteomic profile was excluded after principal-component quality control, leaving 29 profiles (14 DLM and 15 NLM). Two senior orthopedic surgeons independently assessed the radiographic and intraoperative joint status, and two pathologists blinded to group independently graded the meniscal specimens; discrepancies were resolved by consensus. The baseline characteristics are provided in Table 1. The Biomedical Ethics Committee of West China Hospital, Sichuan University, approved the study (2022 Review No. 426). All participants provided written informed consent, and the study complied with the Declaration of Helsinki and relevant institutional regulations.

**TABLE 1:** Baseline characteristics of the study cohorts.

Baseline characteristics	Overall population (n = 187)		Histological analysis (n = 40)		Transcriptomic and proteomic analysis (n = 30)		THBS1 validation by qRT-PCR/WB (n = 12)		Cell-based assay (n = 6)	
	DLM (n = 122)	NLM (n = 65)	DLM (n = 20)	NLM (n = 20)	DLM (n = 15)	NLM (n = 15)	DLM (n = 6)	NLM (n = 6)	DLM (n = 3)	NLM (n = 3)
Demographic characteristics										
Age ( mean ± SD , years)	36.2 ± 17.5	45.9 ± 15.8	35.8 ± 16.2	42.7 ± 17.4	31.4 ± 19.9	50.1 ± 15.8	34.6 ± 18.4	44.7 ± 12.5	24.2 ± 6.5	28.6 ± 4.2
Sex (male/female, N)	37/85	34/31	14/6	15/5	4/11	5/10	3/3	4/2	2/1	2/1
BMI ( mean ± SD , kg/m^2^)	24.7 ± 3.4	22.8 ± 3.9	24.5 ± 5.2	22.9 ± 3.8	22.1 ± 5.0	23.6 ± 2.5	23.2 ± 6.3	22.5 ± 3.5	21.6 ± 2.2	20.8 ± 3.2
Clinical characteristics										
Involved knee (left/right, N)	62/60	32/33	10/10	11/9	8/7	8/7	3/3	3/3	1/2	1/2
Symptoms duration ( mean ± SD *,* months)	28.5 ± 20.4	6.2 ± 5.5	28.0 ± 19.8	6.5 ± 5.3	29.0 ± 20.1	6.0 ± 5.6	27.5 ± 20.0	6.3 ± 5.4	8.6 ± 5.4	3.7 ± 2.5
K–L grade (0/I, N)	85/37	44/21	14/6	15/5	11/4	12/3	5/1	5/1	3/0	3/0
Trauma history (no/yes, N)	92/30	3/62	15/5	1/19	12/3	0/15	3/3	0/6	2/1	0/3
OA family history (no/yes, N)	102/20	56/9	18/2	19/1	13/2	14/1	5/1	5/1	3/0	3/0
Concomitant OA in other joints (no/yes, N)	118/4	63/2	19/1	20/0	15/0	15/0	5/1	6/0	3/0	3/0
Intraoperative joint and meniscal status										
Outerbridge grade (0/I, N)	98/24	52/13	16/4	17/3	13/2	13/2	5/1	5/1	2/1	2/1
Meniscus tear (no/yes, N)	13/109	0/65	2/18	0/20	1/14	0/15	1/5	0/6	0/3	0/3
Meniscal tear pattern (longitudinal/horizontal/transverse/complex, N)	30/48/18/13	28/12/19/6	5/9/3/1	7/6/5/2	4/8/2/0	6/4/4/1	1/3/1/0	2/2/1/1	0/3/0/0	2/0/1/0
Concomitant injuries (cruciate ligament, medial meniscus) (no/yes, N)	105/17	13/52	17/3	2/18	13/2	1/14	5/1	1/5	3/0	0/3

Values are the mean ± standard deviation unless stated otherwise. RNA sequencing included 15 DLM and 15 NLM specimens; quantitative proteomics included 14 DLM and 15 NLM profiles after quality-control exclusion of one DLM sample; BMI, body mass index; DLM, discoid lateral meniscus; K–L, Kellgren–Lawrence; NLM, morphologically normal lateral meniscus; OA, osteoarthritis.

### 2.2 Histology and imaging

Specimens were fixed in paraformaldehyde, paraffin-embedded, and sectioned for H&E, Masson trichrome, Safranin O-fast green, and Sirius Red staining. Meniscal degeneration was graded using the Pauli system (Pauli et al., 2011). For immunofluorescence, antigen retrieval was performed in citrate buffer (pH 6.0) under high pressure for 4 min, followed by serum blocking for 20 min at room temperature. Sections were incubated overnight at 4 °C with primary antibodies against COL1A1 (1:200), COL2A1 (1:200), aggrecan (1:200), TE-7 (1:200), SOX9 (1:100), THBS1 (1:100), integrin α3β1 (1:200), and syndecan-4 (1:200), which were all procured from ABclonal (Wuhan, China). Alexa Fluor-conjugated secondary antibodies (1:300; HuaAn Biotechnology, Hangzhou, China) were applied for 1 h at 37 °C, and nuclei were counterstained with DAPI (Solarbio, Beijing, China) for 4 min–10 min. THBS1 immunohistochemistry used anti-THBS1 (1:100; ABclonal) and an HRP-conjugated goat anti-rabbit secondary antibody (1:1,000; HuaAn Biotechnology). Images were acquired by confocal microscopy under consistent settings. Cellularity was quantified in one 400× TE-7/SOX9 immunofluorescence image per specimen by counting all DAPI-positive nuclei. TE-7-positive/SOX9-negative, TE-7-negative/SOX9-positive, and double-positive cells were classified as fibroblast-like, chondrocyte-like, and fibrochondrocytes, respectively. Counts were reported per analyzed image. COL1A1, COL2A1, and aggrecan signals were quantified in ImageJ as whole-field mean fluorescence intensity under consistent acquisition settings. Tissue scoring and image quantification were performed blinded to the groups. Collagen-fibril diameter and density were measured in transmission electron micrographs, collagen architecture was graded using the TEM collagen meniscal architecture score, and fiber organization and surface topography were examined by scanning electron microscopy.

### 2.3 Transcriptomic analysis

Total RNA was isolated using TRIzol, and samples with RNA integrity number >7.0 were used for library construction. Libraries were sequenced on the BGISEQ-500 platform to generate approximately 20 million 100-bp paired-end reads per sample. Reads were aligned to GRCh38 using HISAT2 (v2.2.1) (Kim et al., 2019), and the expression was quantified using RSEM (v1.3.1) (Li and Dewey, 2011). Differential expression was analyzed using DESeq2 (v1.44.0), with age included as a covariate (Love et al., 2014). Differential genes were defined by absolute fold change >2 and Benjamini–Hochberg false-discovery rate <0.05. Gene Ontology and KEGG pathway enrichment used clusterProfiler (v4.12.6) (Wu et al., 2021; Kanehisa et al., 2025), and candidates were screened against ECM-, cartilage- and bone-related gene sets. Candidate expression patterns were visualized by unsupervised hierarchical clustering.

### 2.4 Proteomic analysis

Proteins were extracted, digested with trypsin, and analyzed on a timsTOF Pro platform. A data-dependent acquisition library and data-independent acquisition quantitative runs were generated using the diaPASEF workflow (Meier et al., 2020). Identification quality was controlled with mProphet (Reiter et al., 2011), and the processed protein-abundance matrix was analyzed using limma (v3.60.6), with age included as a covariate (Ritchie et al., 2015). Proteins with absolute fold change >1.5 and nominal *P* < 0.05 were screened. Both omics datasets were intersected with ECM-, cartilage-, and bone-related gene sets.

### 2.5 THBS1 validation

THBS1 abundance in tissues and cells was measured by quantitative reverse-transcription PCR and Jess Simple Western capillary immunoassay. For Jess analysis, lysates were adjusted to 0.25 μg/μL, separated with the 12 kDa–230 kDa module, probed with anti-THBS1 (1:500; ABclonal) and HRP-conjugated secondary antibody (1:500), detected by chemiluminescence, and quantified in Compass software. Jess signals were exported as Compass virtual-lane views. For the overexpression experiment, 20 µg–30 µg protein was separated by SDS-PAGE, transferred to a PVDF membrane, probed with anti-THBS1 (1:500; ABclonal) and β-actin loading control, detected by enhanced chemiluminescence, and quantified in ImageJ. Complete Jess exports and the uncropped conventional membrane are provided in Supplementary Figure S8. For quantitative PCR, RNA was reverse-transcribed and amplified with SYBR Green using GAPDH as the reference gene. Cycling comprised 40 cycles of 95 °C for 15 s, 60 °C for 30 s, and 72 °C for 30 s. Relative expression was calculated by the 2^−^ΔΔCT method from triplicate reactions; the primer sequences are provided in Supplementary Table S1.

### 2.6 Cell culture and THBS1 manipulation

Primary meniscal cells were isolated by sequential digestion with 0.2% pronase for 1 h and 0.02% collagenase II overnight, cultured in DMEM/F12 with 10% fetal bovine serum and 1% penicillin–streptomycin at 37 °C and 5% CO_2_, passaged at a 1:3 ratio, and used at passages 2–4. Cells from each of three independent NLM donors received GFP-labeled THBS1 short-hairpin RNA or the matched non-targeting vector. Cells from each of three independent DLM donors received an RFP-labeled THBS1-expression vector or the matched empty vector. Lentiviral transduction was performed at a multiplicity of infection of 25 and assessed after 72 h. Reporter-positive fractions were calculated in ImageJ as fluorescent-reporter-positive cells divided by the total cells in the corresponding brightfield image. Recorded fractions were approximately 99.8% for NLM-sh-THBS1 and 99.7% for DLM-OE-THBS1. THBS1 abundance was verified by quantitative PCR and immunoblotting.

### 2.7 Live-cell and migration assays

Area confluence was recorded by IncuCyte live-cell imaging every 3 h for 72 h and normalized to the baseline value of each well. Scratch-wound relative density was recorded every 3 h for 48 h. Transwell migration was assessed using inserts with 8-µm pores, followed by imaging and counting of migrated cells. Each assay used three independent biological replicates, with each intervention compared with its matched vector control.

### 2.8 Three-dimensional meniscal spheroids

NLM-sh-NC, NLM-sh-THBS1, DLM-OE-NC, and DLM-OE-THBS1 cells were aggregated at high density in non-adherent plates and cultured for 4 weeks. Spheroid diameter was measured microscopically. Spheroids were processed for H&E, Masson trichrome and Safranin O-fast green staining; immunohistochemistry for COL1A1, COL2A1, and aggrecan; transmission electron microscopy; and scanning electron microscopy. Collagen and aggrecan staining was quantified as image-level integrated optical density. Fibrillar features in electron micrographs were evaluated qualitatively.

### 2.9 Statistics and reproducibility

Continuous data are reported as the mean ± s.d., with individual biological-replicate points displayed where available; categorical variables are the counts. Normality and homogeneity of variance were assessed using the Shapiro–Wilk and Levene tests, respectively. Two-group comparisons used two-sided Student's t-tests when assumptions were met and Mann–Whitney U-tests otherwise. Multiple groups were analyzed by one-way analysis of variance with Tukey testing or by Kruskal–Wallis testing, as appropriate. Longitudinal live-cell trajectories were compared using two-way repeated-measures analysis of variance with group and time as factors. Categorical variables were compared using χ^2^ or Fisher’s exact tests. The experimental unit and n are defined in each figure legend. No statistical method was used to predetermine the sample size; eligible available specimens defined the clinical cohorts. Tissue scoring and image quantification were performed blinded to groups. No data were excluded except one DLM proteomic profile after prespecified principal-component quality control. Analyses used GraphPad Prism 9 and R 4.4.0; two-sided *P* < 0.05 was considered significant.

## 3 Results

### 3.1 DLM tissue exhibits reduced cellularity and disorganized collagen architecture

Arthroscopic and histological assessment showed abnormal ECM organization in DLM. Pauli macroscopic scores were higher in DLM than in NLM (8.0 ± 1.6 versus 6.3 ± 1.8; mean difference, 1.65; 95% confidence interval (CI), 0.52 to 2.78; *P* = 0.0055; Figures 1a,b and Supplementary Figure S1), and Pauli histological scores were also elevated (12.7 ± 2.6 versus 8.0 ± 2.5; mean difference, 4.70; 95% CI, 3.09 to 6.42; *P* < 0.0001; Figures 1a,c). Representative H&E, Masson trichrome, and Safranin O-fast green images showed a loosened matrix with mucoid and vacuolar changes (Figure 1a). In the TE-7/SOX9 immunofluorescence analysis, DLM had 42% fewer total cells per analyzed image than NLM (21.9 ± 12.4 versus 38.0 ± 10.6; mean difference, −16.10; 95% CI, −23.49 to −8.71; *P* < 0.0001). Fibroblast-like cells were lower in DLM (3.6 ± 2.6 versus 12.6 ± 4.5; mean difference, −9.05; 95% CI, −11.41 to −6.69; *P* < 0.0001), as were chondrocyte-like cells (6.2 ± 2.1 versus 8.6 ± 3.6; mean difference, −2.45; 95% CI, −4.32 to −0.58; *P* = 0.0117) and fibrochondrocytes (11.2 ± 6.8 versus 15.3 ± 4.1; mean difference, −4.15; 95% CI, −7.73 to −0.57; *P* = 0.0242; Figures 1d-g; Supplementary Figure S2). Fibroblast-like cells, consequently, represented a smaller fraction of DLM cells (Figure 1i).

**FIGURE 1:**
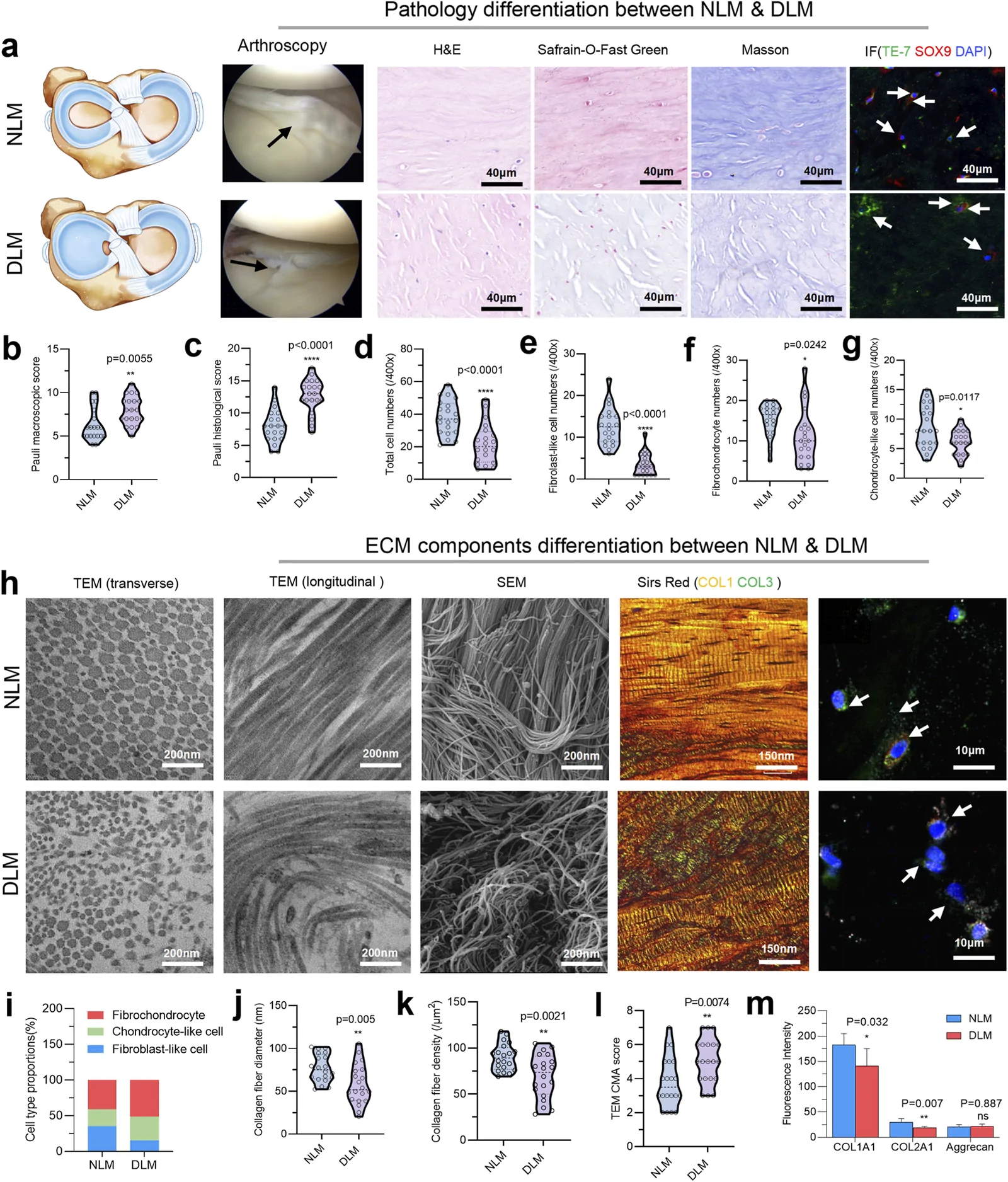
DLM exhibits abnormal extracellular-matrix organization and cellularity compared with NLM. **(a)** Morphological model and representative arthroscopic, hematoxylin and eosin (H&E), Masson trichrome, Safranin O-fast green, and TE-7/SOX9 immunofluorescence images of morphologically normal lateral meniscus (NLM) and discoid lateral meniscus (DLM). H&E panels show tissue morphology; cellularity was quantified from the TE-7/SOX9 immunofluorescence images. Black and white arrows indicate representative morphological or labeled features identified in the panel annotations. Scale bar, 40 µm. TE-7 (green) identifies fibroblast-like cells, SOX9 (red) identifies chondrocyte-like cells, and double-positive cells are classified as fibrochondrocytes. **(b,c)** Pauli macroscopic and histological scores. **(d-g)** Total cells, fibroblast-like cells, fibrochondrocytes, and chondrocyte-like cells per analyzed image. **(h)** Representative transmission electron microscopy (TEM; transverse and longitudinal; scale bars, 200 nm), scanning electron microscopy (SEM; scale bar, 200 nm), Sirius Red polarization (scale bar, 150 µm), and matrix immunofluorescence (scale bar, 10 µm). In the matrix panel, COL1A1 is green, COL2A1 cyan, aggrecan is red, and DAPI is blue; white arrows indicate representative labeled matrix signals. **(i)** Cell-subtype proportions. **(j-l)** Collagen-fibril diameter, fibril density, and TEM collagen meniscal architecture score. **(m)** Whole-field mean fluorescence intensity of COL1A1, COL2A1, and aggrecan. Data are represented as the mean ± s.d.; n = 20 independent patient specimens per group. Two-sided tests were used as described in Methods. *P < 0.05; **P < 0.01; ****P < 0.0001; ns, not significant.

Transmission electron microscopy showed thinner collagen fibrils in DLM (57.9 ± 23.3 versus 76.9 ± 16.2 nm; mean difference, −18.92; 95% CI, −31.76 to −6.07; *P* = 0.005), lower fibril density (69.4 ± 23.8 versus 90.0 ± 14.6 fibrils per µm^2^; mean difference, −20.65; 95% CI, −33.30 to −8.00; *P* = 0.0021), and higher TEM collagen meniscal architecture scores (5.1 ± 1.3 versus 3.8 ± 1.5; mean difference, 1.25; 95% CI, 0.36 to 2.14; *P* = 0.0074; Figures 1h,j-l). Scanning electron microscopy showed disorganized fiber architecture. Whole-field mean fluorescence intensity was lower for type-I collagen (mean difference, −41.60; 95% CI, −78.82 to −4.38; *P* = 0.032) and type-II collagen (mean difference, −10.85; 95% CI, −17.92 to −3.78; *P* = 0.007), whereas aggrecan was unchanged (*P* = 0.887; Figures 1h,m; Supplementary Figure S3). Sirius Red polarization showed fewer thick type-I bundles and more thin type-III fibers in DLM (Supplementary Figure S4).

### 3.2 Cross-omics analyses identify THBS1 as a candidate

RNA sequencing identified 288 differentially expressed genes (57 higher and 231 lower in DLM), and quantitative proteomics identified 316 differentially expressed proteins (247 higher and 69 lower) using the prespecified screening thresholds (Figures 2a,b,e and Supplementary Data 1–4). The central values 14 and 19 in the three-set intersections denote molecules shared by the DLM-versus-NLM differential set, the ECM-related set, and the bone/cartilage-development set (Figures 2c,f). Candidate heatmaps separated the sampled DLM and NLM profiles (Figures 2d,g).

**FIGURE 2:**
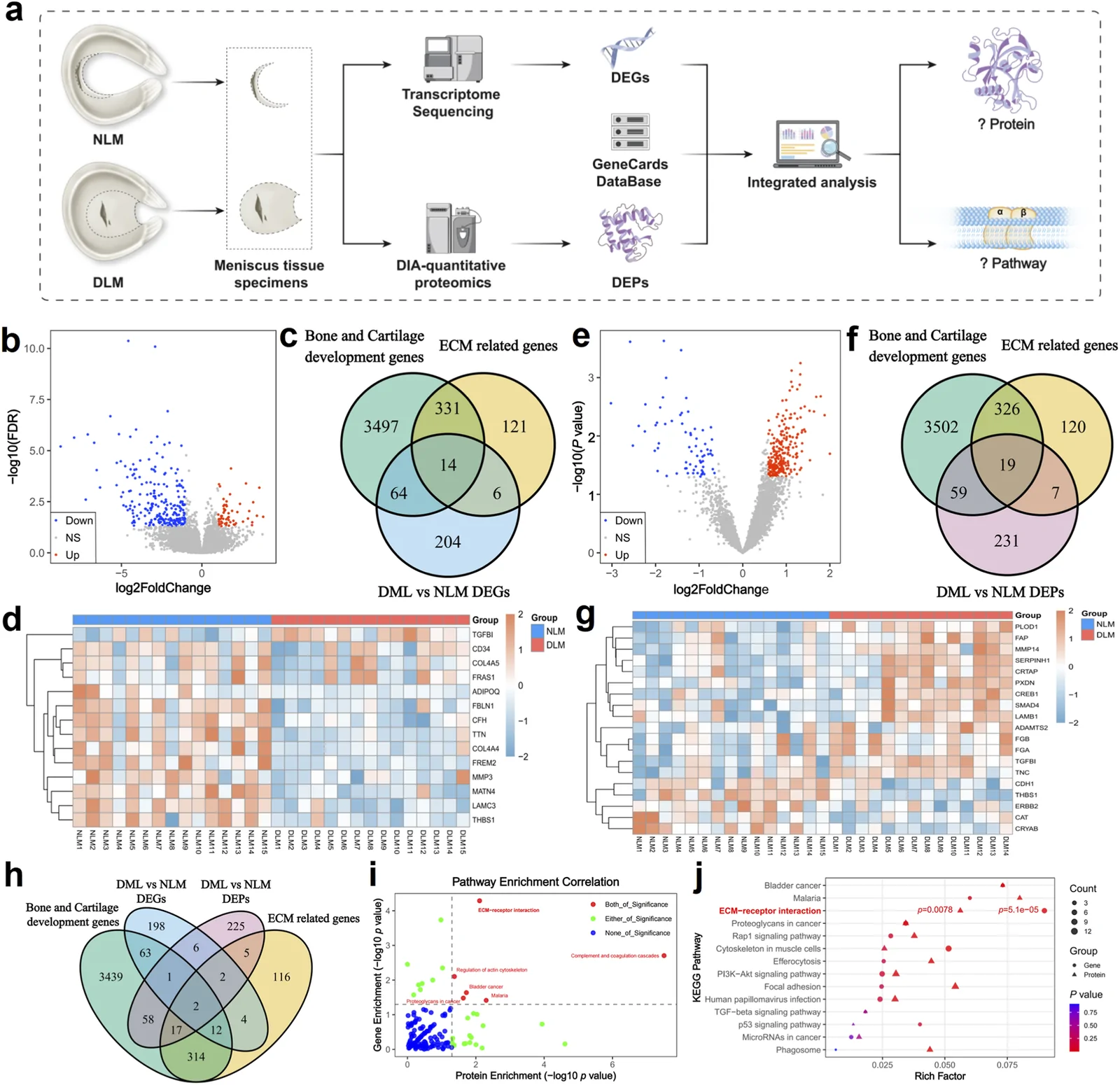
Integrated omics analyses nominate THBS1 and extracellular-matrix receptor interaction in DLM. **(a)** Study workflow. **(b)** RNA volcano plot using absolute fold change >2.0 and adjusted *P* < 0.05. **(c)** Intersection of DLM-versus-NLM differentially expressed genes and extracellular-matrix-related genes and bone/cartilage-development genes; the central value 14 comprises 12 transcriptome-only candidates plus THBS1 and TGFBI. **(d)** Heatmap of the 14 candidates. **(e)** Protein volcano plot using absolute fold change >1.5 and nominal *P* < 0.05. **(f)** Corresponding three-set protein intersection; the central value 19 comprises 17 proteome-only candidates plus THBS1 and TGFBI. **(g)** Heatmap of the 19 candidates. **(h)** Four-set intersection showing THBS1 and TGFBI as cross-omics candidates. TGFBI denotes transforming growth factor-beta-induced protein and is distinct from the TGF-beta ligand. **(i,j)** Cross-omics KEGG enrichment. ECM–receptor interaction was enriched in both datasets. Rhombus, RNA; circle, protein; bubble size, molecule count; color, nominal enrichment *P*-value. RNA sequencing included n = 15 independent specimens per group; quantitative proteomics included n = 14 DLM and n = 15 NLM specimens after quality-control exclusion of one DLM profile.

THBS1 and transforming growth factor-beta-induced protein (TGFBI) were altered at both RNA and protein levels (Figure 2h and Supplementary Data 5). THBS1 was lower in RNA data (log2 fold change, −1.67; adjusted *P* = 0.0108) and protein data (log2 fold change, −2.58; nominal *P* = 0.000244). The protein-level result did not remain significant after dataset-wide adjustment. ECM–receptor interaction was enriched in both datasets (RNA P = 5.15 × 10^−5^; protein *P* = 0.00779; Figures 2i,j and Supplementary Data 2, 4, 6, and 7).

### 3.3 THBS1 abundance is lower in DLM

Independent tissue assays confirmed reduced THBS1 abundance. Jess Simple Western analysis showed a 6.67-fold lower THBS1 protein abundance in DLM than in NLM (95% CI, 5.04 to 8.27; *P* < 0.0001; Figures 3a,b), and quantitative reverse-transcription PCR showed lower THBS1 mRNA (*P* < 0.0001; Figure 3c). Immunohistochemistry showed stronger THBS1 immunoreactivity in NLM, particularly around meniscal cells and adjacent collagen-rich matrix, whereas DLM staining was weaker and patchier (*P* < 0.0001; Figures 3d,e; Supplementary Figure S5). In NLM multichannel images, THBS1 signal was most evident around TE-7/SOX9 double-positive and TE-7-positive cells, whereas all examined cell regions in DLM showed weaker signal. Donor-derived DLM cells also showed less THBS1 fluorescence than NLM cells, with signal in membrane-associated and perinuclear regions (Figure 3f-h; Supplementary Figure S6). Exploratory multichannel images displayed overlapping THBS1, integrin α3β1, and syndecan-4 signal regions (Figure 3h).

**FIGURE 3:**
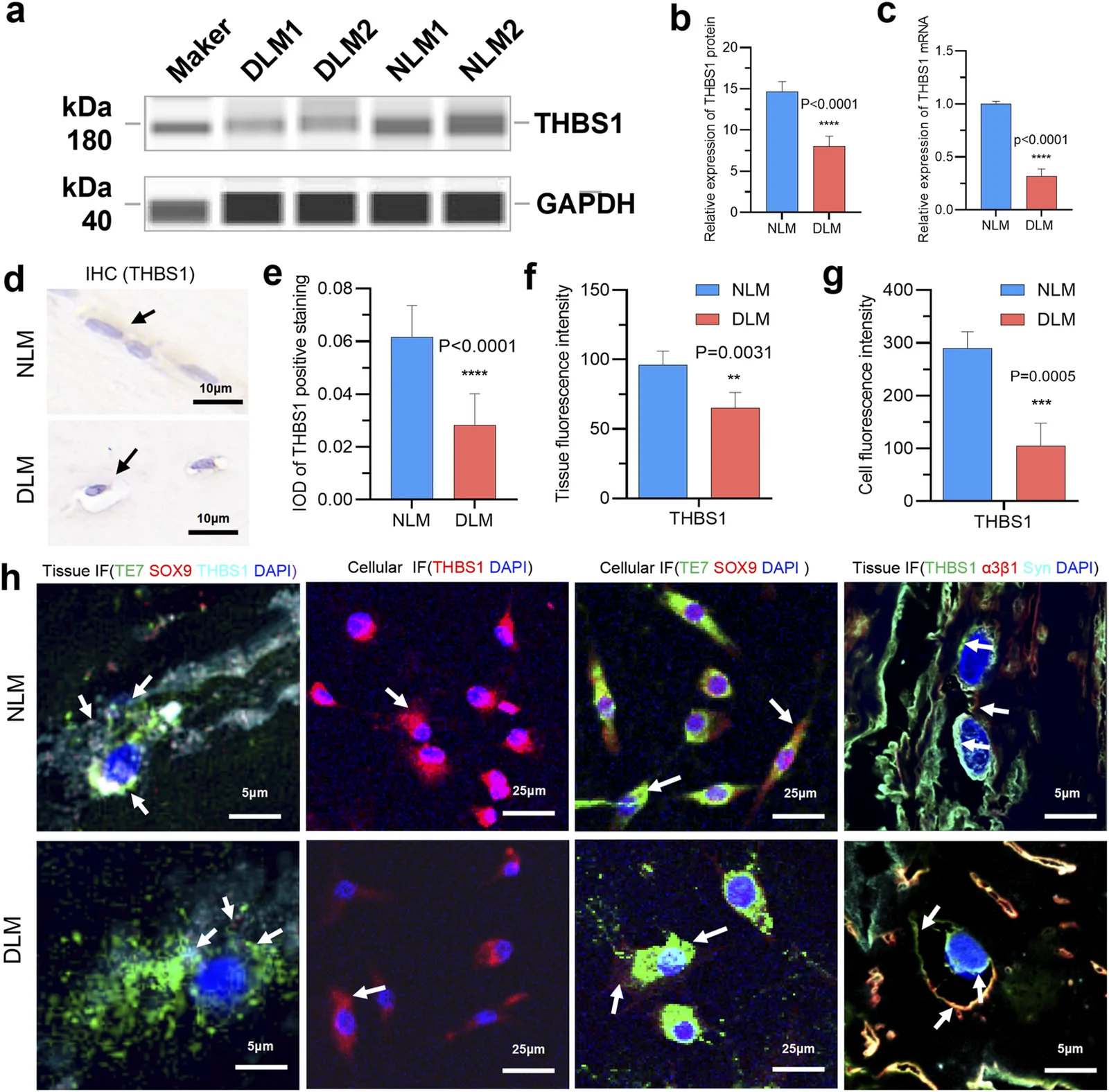
THBS1 abundance is lower in DLM tissue and donor-derived cells. **(a,b)** Jess Simple Western virtual-lane views and tissue THBS1 quantification. The rectangular lanes are Compass software-generated capillary outputs; complete exports are provided in Supplementary Figure S8. **(c)** THBS1 mRNA measured by quantitative reverse-transcription PCR. **(d,e)** THBS1 immunohistochemistry and integrated optical-density quantification; scale bar, 10 μm. **(f,g)** Whole-field THBS1 fluorescence in tissue and primary cells. **(h)** Representative multichannel images: panel 1, tissue THBS1 (cyan), TE-7 (green), and SOX9 (red); panel 2, primary-cell THBS1 (red); panel 3, primary-cell TE-7 (green) and SOX9 (red); and panel 4, tissue syndecan-4 (cyan), THBS1 (green), and integrin α3β1 (red). DAPI is shown in blue. White arrows indicate representative positive fluorescence signals. Scale bars are shown in each image. Data are represented as the mean ± s.d.; n = 6 independent patient specimens per group. Two-sided tests were used as described in Methods. **P < 0.01; ****P < 0.0001.

### 3.4 THBS1 manipulation changes area-confluence trajectories and migration

THBS1 knockdown in NLM-derived cells reduced protein abundance to approximately 38% of the matched control (*P* = 0.0001), whereas THBS1 overexpression in DLM-derived cells increased protein abundance 2.6-fold relative to that of the matched control (95% CI, 2.26 to 3.00; *P* < 0.0001); mRNA changed concordantly (both *P* < 0.0001; Figures 4a-f). The interventions were established in cells from three independent donors per group and compared with within-donor vector controls (Supplementary Figure S7). Representative phase-contrast images showed a more scattered growth pattern after knockdown and denser band-like clusters after overexpression (Figure 4g).

**FIGURE 4:**
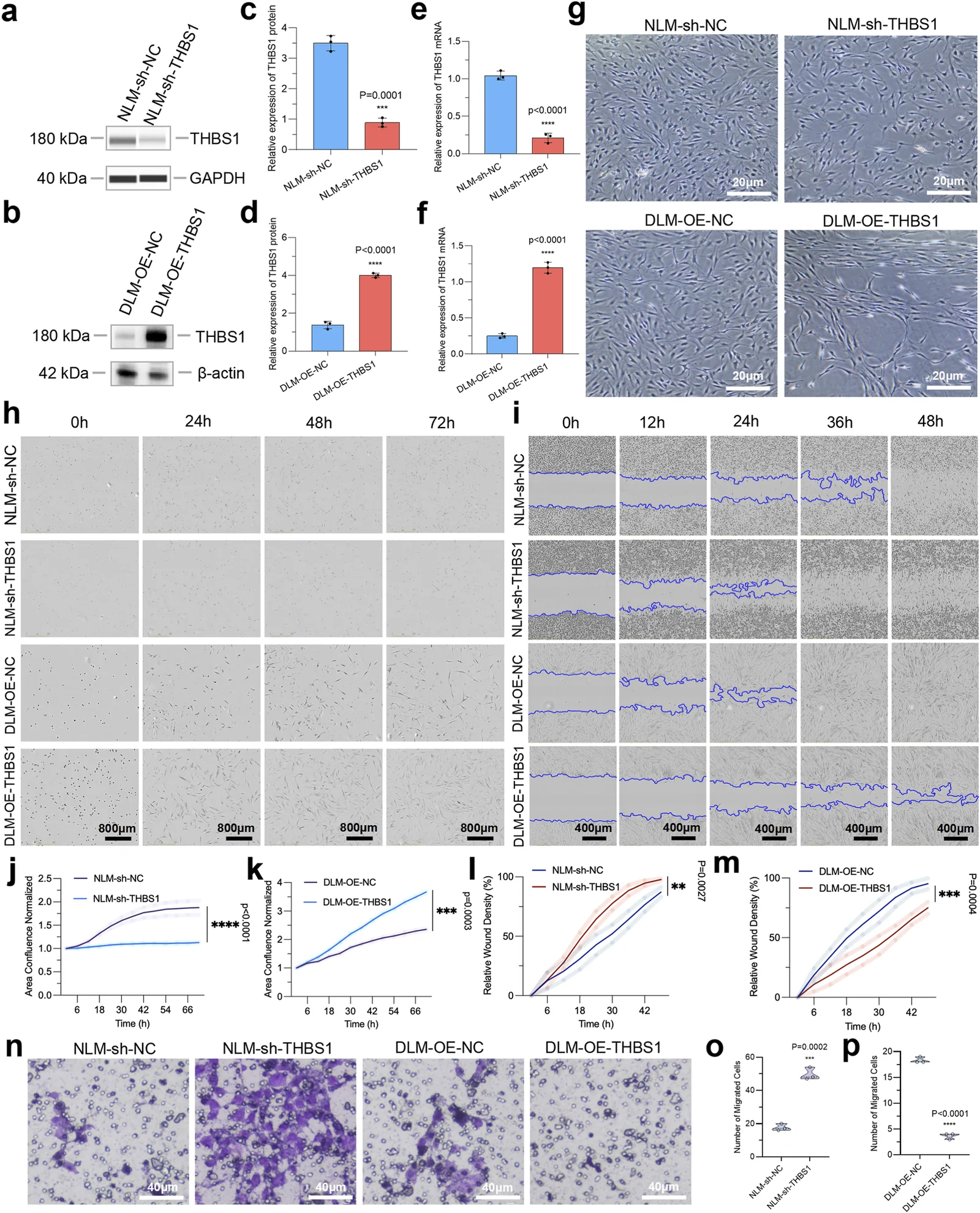
THBS1 manipulation alters area-confluence trajectories and meniscal-cell migration. **(a-d)** THBS1 protein after knockdown in NLM-derived cells or overexpression in DLM-derived cells; a shows a Jess virtual-lane view, and b is conventional Western blotting. **(c,d)** Corresponding quantitative analyses. Complete outputs are provided in Supplementary Figure S8. **(e,f)** THBS1 mRNA. **(g)** Representative phase-contrast culture images for each intervention and matched vector control; scale bar, 20 μm. **(h,j,k)** Representative IncuCyte images and baseline-normalized area-confluence trajectories over 72 h. **(i,l,m)** Representative scratch images and relative wound-density trajectories over 48 h. **(n-p)** Transwell images and migrated-cell counts; scale bar, 40 µm. Longitudinal trajectories were analyzed by two-way repeated-measures analysis of variance for group and time; each intervention was compared with its matched control. Data are represented as the mean ± s.d. or mean with plotted uncertainty, as shown; n = 3 independent biological replicates. ***P < 0.001; ****P < 0.0001.

Live-cell imaging showed a lower baseline-normalized area-confluence trajectory after THBS1 knockdown (mean difference, −48.3%; 95% CI, −65.4 to −31.1; *P* < 0.0001) and a higher trajectory after THBS1 overexpression (mean difference, 61.6%; 95% CI, 34.6 to 88.5; *P* = 0.0003; Figures 4h,j,k). Scratch-wound relative density was higher after knockdown (*P* = 0.0077) and lower after overexpression (*P* = 0.0004; Figures 4i,l,m). Transwell assays showed more migrated cells after knockdown (*P* = 0.0002) and fewer migrated cells after overexpression (*P* < 0.0001; Figures 4n-p).

### 3.5 THBS1 manipulation changes collagen-rich matrix in spheroids

High-density meniscal cells formed three-dimensional spheroids during 4 weeks of culture. NLM-sh-THBS1 spheroids were approximately 26% smaller than matched NLM-sh-NC spheroids (mean diameter difference, −140.0 µm; 95% CI, −174.6 to −105.4; *P* = 0.0004). DLM-OE-THBS1 spheroids were approximately 17% larger than DLM-OE-NC spheroids (mean difference, 89.0 µm; 95% CI, 57.3 to 120.7; *P* = 0.0015; Figures 5a,b). Histological staining showed less collagen-rich matrix after knockdown and more collagen-rich matrix after overexpression (Figure 5c).

**FIGURE 5:**
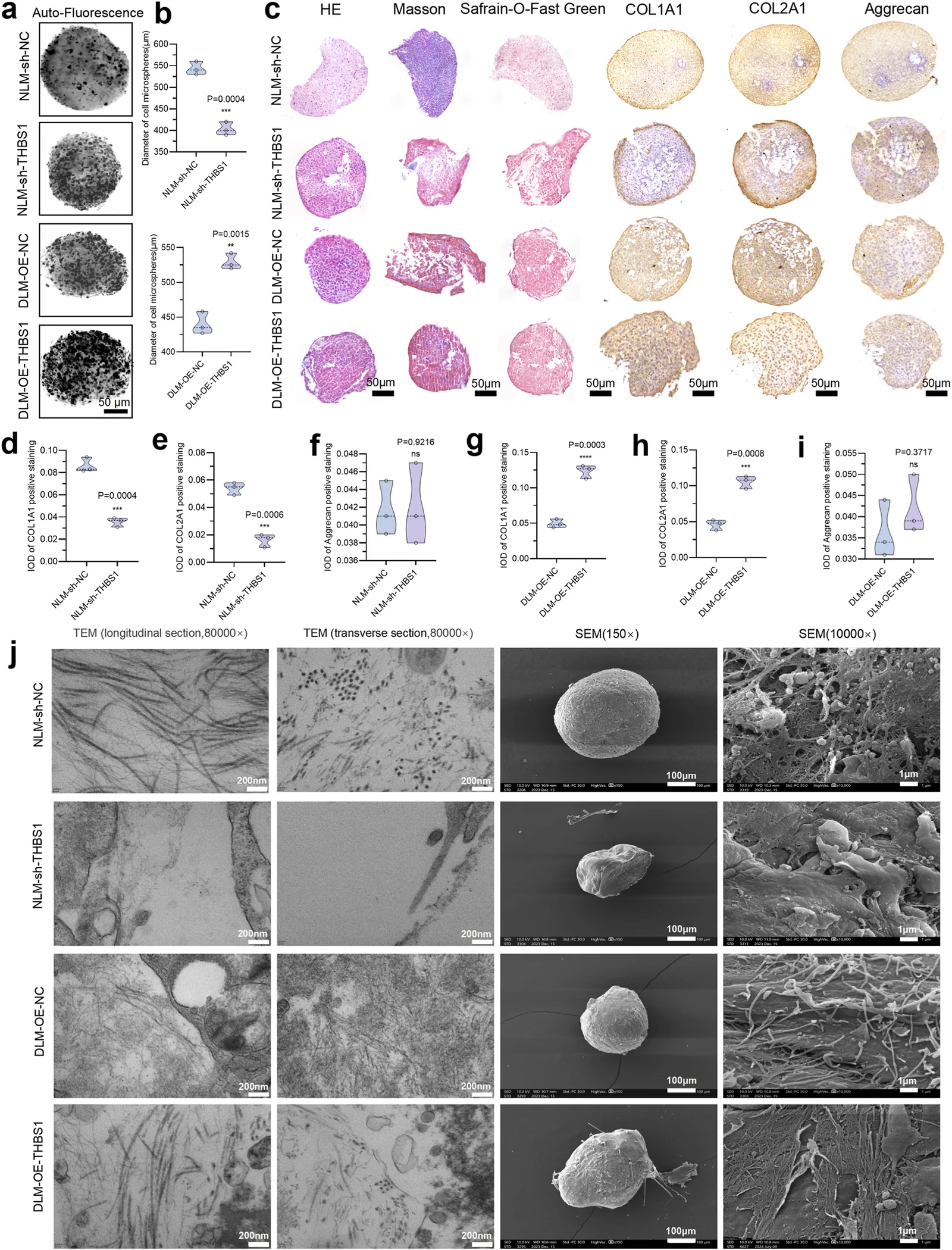
THBS1 manipulation alters collagen-rich matrix in three-dimensional meniscal spheroids. **(a,b)** Diameter of high-density spheroids generated from NLM-sh-THBS1 and DLM-OE-THBS1 cells after 4 weeks; scale bar, 50 μm. **(c)** H&E, Masson trichrome, Safranin O-fast green, COL1A1, COL2A1, and aggrecan staining. **(d-i)** Integrated optical-density quantification of COL1A1 **(d,g)**, COL2A1 **(e,h)**, and aggrecan **(f,i)**. Collagen integrated optical density was not normalized to cell content. **(j)** Representative transmission electron microscopy (TEM) images labeled as longitudinal and transverse sections and scanning electron microscopy (SEM) images; magnifications and scale bars are shown within each micrograph. Fibrillar organization in these images was assessed qualitatively. Data are represented as the mean ± s.d.; n = 3 independent biological replicates per group. Two-sided tests were used as described in Methods. **P < 0.01; ***P < 0.001; ns, not significant.

THBS1 knockdown reduced type-I and -II collagen staining to 30.2%–40.3% of the matched-control levels (*P* = 0.0004 and *P* = 0.0006) without a detected aggrecan difference (*P* = 0.9216; Figures 5d-f). THBS1 overexpression increased type-I and -II collagen staining 2.3–2.5-fold (*P* = 0.0003 and *P* = 0.0008), again without a detected aggrecan difference (*P* = 0.3717; Figures 5g-i). In the representative electron micrographs, the NLM-sh-THBS1 field showed near-complete loss of recognizable fibrillar architecture. DLM-OE-THBS1 spheroids showed denser and more organized fibrillar features than DLM-OE-NC spheroids (Figure 5j).

## 4 Discussion

The principal finding is that reduced THBS1 accompanies the depleted and disorganized collagen-rich matrix of symptomatic DLM and influences the related phenotypes in donor-derived models. Histology and ultrastructure showed fewer cells per analyzed image and altered collagen architecture, cross-omics screening nominated THBS1, and independent assays confirmed lower abundance. Reciprocal perturbation changed area-confluence trajectories, migration, spheroid size, collagen staining, and qualitative fibril organization. Together, these observations support a functional contribution of THBS1 to collagen-rich matrix organization *in vitro*, but they do not establish THBS1 as the primary cause of DLM or as a fibrillogenesis-specific regulator.

The matrix findings were more consistent for collagen than for aggrecan. DLM tissue and THBS1-deficient spheroids showed lower collagen signals and disorganized fibrillar features, whereas aggrecan was comparatively preserved. The meniscus depends on a regionally organized collagen network, principally circumferential type-I collagen with radial tie fibers and a smaller proteoglycan-rich inner matrix, to resist tensile and compressive loading (Kuczyński et al., 2025; Pasiński et al., 2023). Type-III collagen also regulates fibril structure and biomechanics in cartilage and meniscus (Wang et al., 2020). However, tissue fluorescence and spheroid integrated optical density were not normalized to the cell content, and spheroid electron microscopy was assessed qualitatively. The present experiments, therefore, do not distinguish changes in cell number or total matrix production from a specific defect in fibril assembly.

The spheroid experiments extend the tissue association to perturbation evidence. Depleting THBS1 in NLM-derived cells and overexpressing it in DLM-derived cells produced reciprocal changes across two-dimensional assays and 4-week spheroids. Each of the three donors in a group contributed both intervention and matched-control cultures, thus reducing within-background donor variability. Nevertheless, knockdown was performed only in the NLM background and overexpression only in the DLM background. This asymmetric, non-factorial design and the small donor cohort do not fully separate the effect of THBS1 from other differences between NLM- and DLM-derived cells. In addition, the knockdown magnitude cannot be quantitatively equated with the THBS1 difference in clinical tissue because the tissue and cell experiments used different donor sets and normalization references.

Developmental, biomechanical, and familial factors have long been proposed for DLM (Turati et al., 2021; Campbell et al., 2023; Smillie, 1948; Kaplan, 1957; DASHEFSKY, 1971; de Lambilly et al., 1991). The present cross-sectional comparison cannot determine whether reduced THBS1 and the matrix phenotype are intrinsic to discoid morphology, arise secondarily from chronic abnormal loading and degeneration, or reflect both processes. Both cohorts contained symptomatic operative tissue, the NLM group had traumatic tears, and the groups differed in symptom duration and trauma history. Prior single-cell analysis showed DLM cell populations with altered collagen and matrix-degrading programs (Sun et al., 2022). Our perturbation results show that changing THBS1 can modify matrix-related phenotypes in donor-derived models, but they do not establish the chronology of THBS1 reduction in patients.

THBS1 regulates ECM organization without serving primarily as a structural matrix component (Murphy-Ullrich, 2019; Tan and Lawler, 2009; Adams, 2024). It binds collagens and lysyl oxidase precursors and can support collagen cross-linking in other systems (Tan and Lawler, 2009; Rosini et al., 2018). Loss of other collagen-associated matrix regulators, including decorin and lumican, produces abnormal fibril morphology and tissue fragility (Danielson et al., 1997; Chakravarti et al., 1998), and thrombospondin-2 has been linked to collagen fibrillogenesis in several connective tissues (Calabro et al., 2014; Kyriakides et al., 1998). The spheroid findings are consistent with an organizing contribution of THBS1 to meniscal collagen-rich matrix, but direct assembly kinetics, collagen cross-linking, and quantitative fibril-order measurements were not obtained. Integrins and syndecans are plausible candidates for future matrix-receptor studies (Murphy-Ullrich, 2019; Tan and Lawler, 2009; Berdiaki et al., 2024; Katoh, 2024), but fluorescence overlap in this study does not demonstrate colocalization, physical binding, receptor activation, or downstream signaling.

Clinically, saucerization and meniscal repair improve the shape and mechanics but do not determine the biological origin of the matrix phenotype. Biological augmentation is increasingly being considered in meniscus-preservation strategies (Chen et al., 2024), providing a rationale for future matrix-supportive studies after biomechanical correction. Such translation requires independent donor cohorts, mechanically loaded models, factorial THBS1 perturbation, and direct evaluation of collagen assembly and receptor-dependent signaling before therapeutic conclusions can be drawn.

This study has several limitations. Both cohorts comprised symptomatic operative tissue, and NLM samples came from traumatic tears rather than healthy menisci. The cross-sectional design and between-group differences in age, symptom duration, and trauma history prevent separation of intrinsic DLM biology from secondary effects of loading and degeneration. The archived image record did not retain calibrated field area or sufficiently precise anatomical sub-region, so cellularity is reported per analyzed 400× image rather than as tissue-wide density. The omics cohorts were modest, the proteomic screen used a nominal *P*-value threshold, and one DLM proteomic sample was excluded after quality control. Cell experiments involved three donors per group and used NLM knockdown and DLM overexpression rather than a complete factorial design. Area confluence is influenced by the cell number, projected area, attachment, and survival; BrdU/EdU incorporation, MTT-type viability assays, and dedicated spreading or attachment assays were not performed. Accordingly, area confluence should not be interpreted as a direct measure of proliferation, viability, cell size, spreading, or attachment. Spheroid collagen staining was not normalized to the cell content, fibril organization was assessed qualitatively without a quantitative order metric, and the model lacked the mechanical environment of the knee. Finally, fluorescence overlap did not test receptor binding or downstream signaling.

## 5 Conclusion

Symptomatic DLM was associated with reduced THBS1 abundance and a depleted, disorganized collagen-rich matrix. Reciprocal THBS1 perturbation in donor-derived cells and spheroids supports a functional contribution to cell-migration and matrix phenotypes *in vitro*, while neither establishing that THBS1 initiates DLM pathology nor demonstrating a fibrillogenesis-specific mechanism. Mechanically loaded, factorial models and independent clinical cohorts are needed to define the chronology, mechanism, and translational relevance.

## Data Availability

The datasets presented in this study can be found in online repositories. The names of the repository/repositories and accession number(s) can be found in the article/Supplementary Material.
