# Supplementary material for: Reduced thrombospondin-1 is associated with altered collagen-rich matrix organization in discoid lateral meniscus
Source: Front Cell Dev Biol. 2026 Sep 30;14:1958871. doi: 10.3389/fcell.2026.1958871 (PMC13662068; doi:10.3389/fcell.2026.1958871)
Supplement: Supplementary file 1 [file Supplementaryfile1.docx]

Supplementary Material

# 1 Contents

Supplementary Figures 1-8 and Supplementary Table 1 are included in this file. Supplementary Data 1-7 are supplied separately as machine-readable CSV or XLSX files.

**Supplementary Figure 1.** Arthroscopic appearances of NLM and DLM. NLM shows comparatively mild surface change and a lower Pauli macroscopic score; DLM shows more marked degeneration and a higher score.

**Supplementary Figure 2.** Meniscal-cell immunofluorescence in tissue sections. TE-7 is green, SOX9 red and DAPI blue. Merged images show TE-7-positive, SOX9-positive and double-positive cell populations. Quantification is reported in Fig. 1.

**Supplementary Figure 3.** Matrix immunofluorescence in NLM and DLM sections. COL1A1 is green, COL2A1 cyan, aggrecan red and DAPI blue. White arrows indicate representative labelled matrix signals. Quantification in Fig. 1 is whole-field mean fluorescence intensity and was not calculated per cell or normalized to DAPI-positive nuclei.

**Supplementary Figure 4.** Sirius Red polarization in NLM and DLM. Type I collagen appears yellow-orange and type III collagen green. DLM shows fewer thick type I bundles and more thin, disorganized type III fibres.

**Supplementary Figure 5.** THBS1 immunohistochemistry in independent NLM and DLM specimens. Column numbers denote representative specimens and not matched pairs. Brown immunoreactivity indicates THBS1; nuclei are counterstained blue. Group quantification is reported in Fig. 3.

**Supplementary Figure 6.** Immunofluorescence of primary NLM- and DLM-derived cells after 48 h. TE-7 is green, SOX9 red and DAPI blue; double-positive cells appear yellow.

**Supplementary Figure 7.** Lentiviral reporter imaging 72 h after transduction at a multiplicity of infection of 25. GFP identifies the NLM-sh-THBS1 construct and RFP the DLM-OE-THBS1 construct; corresponding brightfield and merged images are shown. ImageJ estimates were calculated as reporter-positive cells divided by total cells in the corresponding brightfield image and were approximately 99.8% and 99.7%, respectively. THBS1 knockdown and overexpression were validated independently by protein and mRNA measurements in Fig. 4. Scale bars are visible in each panel.

**Supplementary Figure 8.** Complete Jess Simple Western digital exports and an uncropped conventional Western blot membrane. (a) Complete virtual-lane views from two Jess Simple Western analyses of DLM and NLM tissues, showing THBS1, GAPDH and the full molecular-mass ranges. (b) Complete virtual-lane views from Jess analysis of primary NLM cells transduced with a non-targeting control vector (NLM-sh-NC) or THBS1 shRNA (NLM-sh-THBS1), showing THBS1, GAPDH and the full molecular-mass range. GAPDH was used as the loading control for all Jess analyses. Jess is a capillary-based immunoassay, and the rectangular lanes shown in (a) and (b) are Compass software-generated virtual-lane outputs rather than cropped membrane strips. (c) Full uncropped membrane image from conventional SDS–PAGE Western blot analysis of primary DLM cells transduced with an empty control vector (DLM-OE-NC) or a THBS1-expression vector (DLM-OE-THBS1). β-actin was used as the loading control. Molecular masses are indicated in kilodaltons (kDa).

**Supplementary Table 1. Primer sequences used for quantitative reverse-transcription PCR**

| **Gene** | **Forward (5′-3′)** | **Reverse (5′-3′)** |
| --- | --- | --- |
| THBS1 | CCTGTGGAGGAGGGGTACAG | TGTAACCAGGGGGACAAGCA |
| GAPDH | ACAACTTTGGTATCGTGGAAGG | GCCATCACGCCACAGTTTC |
